# Supplementary material for: Dynamic transcriptomic profiles of zebrafish gills in response to zinc supplementation
Source: BMC Genomics. 2010 Oct 11;11:553. doi: 10.1186/1471-2164-11-553 (PMC3091702; doi:10.1186/1471-2164-11-553)
Supplement: Additional file 2 — Interactive Direct Interaction Network representing the molecular interactions between zinc, copper, iron, calcium and proteins encoded by transcripts changed by zinc supplementation. Mini web-site containing index.html and hyperlinked pages in subdirectory describing a Direct Interaction Network automatically generated based on curated interactions contained within the proprietary PathwayArchitect database. Ovals represent proteins and the circles symbolize metal ions. Objects are coloured by their abundance in zebrafish at the time-point they were significantly different from the control is a scale from -4 fold (dark green) to +4 fold (dark red). Where significant differences were found at more than one time-point, the colour overlay shows expression at the first instance. Dark blue squares denote 'binding', and light blue squares 'expression'; green squares stand for 'regulation', green diamonds for 'metabolism', and green circles for 'promoter binding'. Arrow heads indicate directionality of the interaction where annotated. All nodes and edges can be further interrogated by selecting the relative area of the image. [file 1471-2164-11-553-S2.zip › PathwayArchitect Zn xs DIN/96974.html]

# PROTEIN: MMP13

|  |  |
| --- | --- |
| Name | MMP13 |
| Type | PROTEIN |
| Description | matrix metallopeptidase 13 (collagenase 3) |
| Note | Proteins of the matrix metalloproteinase (MMP) family are involved in the breakdown of extracellular matrix in normal physiological processes, such as embryonic development, reproduction, and tissue remodeling, as well as in disease processes, such as arthritis and metastasis. Most MMP's are secreted as inactive proproteins which are activated when cleaved by extracellular proteinases. The protein encoded by this gene cleaves type II collagen more efficiently than types I and III. It may be involved in articular cartilage turnover and cartilage pathophysiology associated with osteoarthritis. The gene is part of a cluster of MMP genes which localize to chromosome 11q22.3. |
| Alias | MMP13 |
|  | matrix metalloproteinase 13 |
|  | matrix metalloproteinase 13 (collagenase 3) |
|  | MMP-13 |
|  | CLG3 |
|  | Matrix metalloproteinase-13 |
|  | collagenase-1 |
|  | Collagenase-3 |
|  | collagenase-3 |
|  | collagenase 3 |
|  | UMRCASE |
|  | Mmp13 |
|  | Mmp1 |
|  | interstitial collagenase |
|  | Clg |
|  | Fragment |


---

|  |  |
| --- | --- |
| GO Component | extracellular space |
|  | extracellular matrix (sensu Metazoa) |


---

|  |  |
| --- | --- |
| GO ID | GO:0005615 |
|  | GO:0008233 |
|  | GO:0004222 |
|  | GO:0051216 |
|  | GO:0000270 |
|  | GO:0016787 |
|  | GO:0008237 |
|  | GO:0004232 |
|  | GO:0008133 |
|  | GO:0008270 |
|  | GO:0030282 |
|  | GO:0005578 |
|  | GO:0046872 |
|  | GO:0006508 |
|  | GO:0005509 |
|  | GO:0030574 |


---

|  |  |
| --- | --- |
| MIM | MIM:600108 |


---

|  |  |
| --- | --- |
| Connectivity | 1105 |


---

|  |  |
| --- | --- |
| Entrez ID | 4322 |
|  | 171052 |
|  | 17386 |


---

|  |  |
| --- | --- |
| Agilent ID | A\_53\_P101752 |
|  | A\_53\_P126310 |
|  | A\_53\_P178614 |
|  | A\_53\_P115157 |
|  | A\_42\_P606126 |
|  | A\_14\_P108910 |
|  | A\_14\_P121786 |
|  | A\_51\_P184484 |
|  | A\_23\_P138931 |


---

|  |  |
| --- | --- |
| Cellular Localization | Extracellular matrix |
|  | Extracellular region |


---

|  |  |
| --- | --- |
| Pathway | Zn xs inventory |
|  | Zn xs DIN |


---

|  |  |
| --- | --- |
| GO Process | proteolysis and peptidolysis |
|  | proteolysis |
|  | bone mineralization |
|  | collagen catabolism |
|  | peptidoglycan metabolism |
|  | cartilage development |


---

|  |  |
| --- | --- |
| UniGene | Hs.2936 |
|  | Rn.10997 |
|  | Mm.5022 |


---

|  |  |
| --- | --- |
| Affymetrix Probeset ID | 100484\_at |
|  | 1388204\_at |
|  | 1417256\_at |
|  | 205959\_at |
|  | 39632\_at |
|  | g13027796\_3p\_at |
|  | M60616\_at |
|  | X66473\_s\_at |
|  | X75308\_at |
|  | RC\_N69322\_s\_at |


---

|  |  |
| --- | --- |
| EC Number | EC 3.4.24.- |


---

|  |  |
| --- | --- |
| GO Function | hydrolase activity |
|  | metallopeptidase activity |
|  | peptidase activity |
|  | interstitial collagenase activity |
|  | metalloendopeptidase activity |
|  | zinc ion binding |
|  | collagenase activity |
|  | metal ion binding |
|  | calcium ion binding |


---

|  |  |
| --- | --- |
| Nucleotide | AY101357 |
|  | XM\_343345 |
|  | NM\_008607 |
|  | AK151626 |
|  | AY135636 |
|  | X82000 |
|  | AK155188 |
|  | X81334 |
|  | BC067522 |
|  | AK222748 |
|  | AY622973 |
|  | M60616 |
|  | AY256443 |
|  | BC074808 |
|  | AK160819 |
|  | X66473 |
|  | AK152916 |
|  | X81640 |
|  | AK153458 |
|  | AK152507 |
|  | X75308 |
|  | AK150728 |
|  | AY256444 |
|  | AY741163 |
|  | BC067523 |
|  | BC074807 |
|  | AK159921 |
|  | NM\_002427 |


---

|  |  |
| --- | --- |
| Protein | CAA57108 |
|  | BAE32010 |
|  | CAA57526 |
|  | CAA47102 |
|  | BAE31273 |
|  | BAD96468 |
|  | BAE33104 |
|  | AAH74807 |
|  | BAE29805 |
|  | CAA57296 |
|  | AAU13907 |
|  | AAH74808 |
|  | AAH67523 |
|  | CAA53056 |
|  | BAE30561 |
|  | P45452 |
|  | NP\_002418 |
|  | NP\_032633 |
|  | BAE36033 |
|  | BAE35483 |
|  | XP\_343346 |
|  | AAP78940 |
|  | AAA72124 |
|  | AAP78939 |
|  | AAT46404 |
|  | BAE31593 |
|  | AAM51172 |
|  | P23097 |
|  | P33435 |
|  | AAH67522 |


---

|  |  |
| --- | --- |
| Organism | Mammal |


---

|  |  |
| --- | --- |
| Location | chromosome 9, 9 A1-A2 (Mus musculus) |
|  | chromosome 8, 8q11 (Rattus norvegicus) |
|  | chromosome 11, 11q22.3 (Homo sapiens) |


---

|  |  |
| --- | --- |
